# Invasive mosquitoes in the non-endemic German region of Saarland: What does the public know and how does it respond?

**DOI:** 10.1016/j.onehlt.2026.101491

**Published:** 2026-06-20

**Authors:** Kamran Dousti, Jakob Kolleck, Irena Harsch, Michael Altmoos, Ina Steinert, Mark Alexander Eichelmann, Phillipp Jung, Sören L. Becker, Sophie Schneitler

**Affiliations:** a Institute of Medical Microbiology and Hygiene, Saarland University, Homburg, Germany; b Zoo Saarbrücken, Saarbrücken, Germany; c Ministry of Labour, Social Affairs, Women and Health, Saarbrücken, Germany; d Ministry of the Environment, Climate, Mobility, Agriculture and Consumer Protection, Saarbrücken, Germany; e Neunkirchen Zoo, Neunkirchen, Germany; f Institute for Medical Microbiology, Immunology and Hygiene, University Hospital Cologne, Cologne, Germany

**Keywords:** Invasive mosquitoes, *Aedes albopictus*, Public knowledge, Preventive behavior, Vector surveillance

## Abstract

By assessing public awareness, preventive behavior, and knowledge regarding invasive mosquito species in Saarland, Germany, a non-endemic region at increasing invasion risk, this study aimed to explore how the introduction of invasive mosquitoes can be addressed in non-endemic areas.

A population-based anonymous questionnaire in Saarland was conducted from February to June 2024. Participants were recruited through broad public dissemination using digital and analog approaches. Nine items assessed mosquito protection practices, knowledge of invasive species, and potential for pathogen transmission.

Among 1064 respondents, 623 (58.6%) met the inclusion criteria. While over half of them recognized stagnant water removal as an effective preventive measure, fewer than one fifth practiced it regularly. Travel medicine consultations were associated with greater use of protective clothing. Among participants aged 26–35, men were more likely than women to report no prior engagement with mosquito prevention measures. Although most participants had heard of *Aedes albopictus*, fewer than half linked it to chikungunya transmission.

Marked discrepancies persist between general awareness and evidence-based preventive behavior.

Targeted seasonal education campaigns represent a modifiable lever to delay vector establishment and improve outbreak preparedness.

## Highlights

• *Aedes albopictus* often known, but limited awareness of disease transmission risk.
• False assumptions about mosquito activity lead to insufficient daytime protection.
• Source reduction is known but not part in routine despite ongoing vector breeding risk.
• Educational deficits in new areas enable outbreaks via unnoticed vector expansion.
• Pre-travel consultations significantly increase adherence to personal protection.

## Graphical abstract

**Figure:**
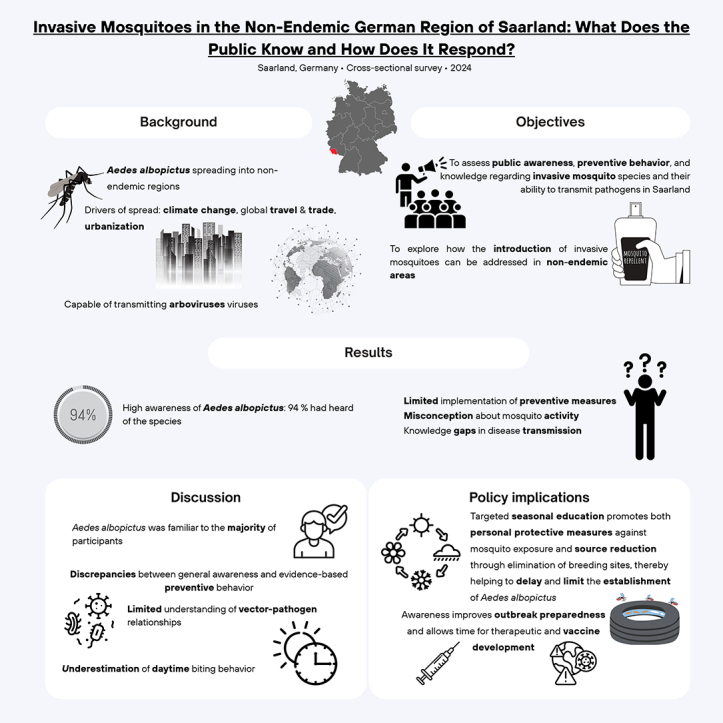

## 1 Introduction

Vector-borne diseases transmitted by vectors, such as the invasive Asian tiger mosquito (*Aedes albopictus*), are among the most significant 21st-century global health challenges. Originally confined to tropical and subtropical zones, these diseases are increasingly emerging in non-endemic regions. Dengue alone accounted for over 14 million cases and more than 11,000 deaths in 2024 [1]. Europe has experienced a concerning increase in both imported and autochthonous cases [2], with *Aedes albopictus* now established in at least 14 European Union countries, including France, Spain, Portugal, and Germany [3]. In 2024, Italy reported 213 locally dengue cases, a European annual record [4]. These trends demonstrate the combined pressures of climate change [5], globalization, and urbanization [6], which together accelerate the geographic expansion of invasive vectors.

Several interacting factors explain the successful establishment of *Aedes albopictus* in new regions. Global air transport, which recovered pre-pandemic levels in 2024 and international maritime shipping systems enable dissemination of different life stages into non endemic areas [7]. Once introduced, the species thrives in urban habitats [8], benefits from extended breeding seasons driven by rising temperatures [9], and possesses life-cycle adaptations such as cold-resistant eggs that facilitate year-round survival [10]. In Germany, a temperature rise of 1.6 °C between 1881 and 2021 [11] has coincided with the species confirmed presence in 6 of 16 federal states [12]. Urbanization further amplifies these dynamics: by 2030, 80% of Europe's population will reside in cities, where high-density housing and urban heat island effects create optimal breeding conditions [11]. The biological profile of *Aedes albopictus*, including daytime biting, oviposition in microhabitats, and rapid spread via human transport networks [13], further increases this risk.

While *Aedes albopictus* is primarily a nuisance biter, the clinical risk emerges when it acts as a vector for arboviruses such as dengue, chikungunya, and Zika [14]. Effective prevention therefore depends not only on surveillance and vector control, but also on informed community behavior. Yet a recent review of knowledge, attitudes, and practices (KAP) related to mosquitoes across eight European countries identified substantial public awareness gaps [15]. The included studies were largely restricted to niche contexts, such as a 2017 German study examining *Anopheles plumbeus* among citizen scientists, and do not reflect the markedly changed distribution landscape of invasive species since then. In Saarland, these gaps are particularly relevant as cross-border movement from France, where *Aedes albopictus* is well established, combined with a climate increasingly favorable to the species, creates elevated invasion risk.

Population-based surveys are a widely used and appropriate method to systematically assess KAP in settings where baseline data are scarce. This study therefore investigated awareness, preventive behaviors, and knowledge of invasive species, particularly *Aedes albopictus*, through a representative cross-sectional survey in a non-endemic region. We aimed to identify knowledge gaps and behavioral patterns to inform public health interventions.

## 2 Methods

A population survey in German was conducted between February and June 2024 in Saarland, a federal state in Germany, bordering France and Luxembourg, with approximately 1 million inhabitants [16]. The survey was made available in both digital and printed formats to maximize accessibility. Both versions were self-administered and completed anonymously. The online questionnaire (Microsoft Forms, Washington, USA) was distributed via websites of Saarland University Medical Center, the Forum magazine, and the Saarbrücken Zoo website, as well as through local radio broadcasts and regional television coverage by “Saarländischer Rundfunk” (SR). Physical outreach involved posters and flyers in botanical and zoological gardens, medical waiting rooms, regional public transport, and the university library, as well as an information stand during the Species Conservation Day on April 1, 2024, at Saarbrücken Zoo, where completed survey forms could subsequently be returned via designated collection boxes.

The survey was divided in two parts. The first section “demographics” included age, gender, and postal code. The main section of the survey included nine questions (6× single choice matrix questions, 3× single choice questions) assessing participants' experiences, knowledge, and beliefs related to mosquito protection and awareness of *Aedes albopictus*. Topics ranged from personal use of protection methods and perceived effectiveness (Questions (Q) 1–3), to ecological concerns (Q4), awareness of invasive species (Q5–7), pathogen transmission (Q8), and timing of mosquito activity (Q9) (see supplementary material). To evaluate the comprehensibility of the questionnaire, a preliminary version was disseminated to 16 individuals with varying degrees of education, age, gender and with no prior familiarity with the study topic.

Data were descriptively analyzed in Microsoft® Excel® (Microsoft 365, Redmond, United States of America) and subjected to statistical evaluation in GraphPad Prism (GraphPad Software, San Diego, United States of America). Group comparisons were conducted using the Mann–Whitney *U* test, with statistical significance defined as *p* < 0.05.

**Fig. 1:**
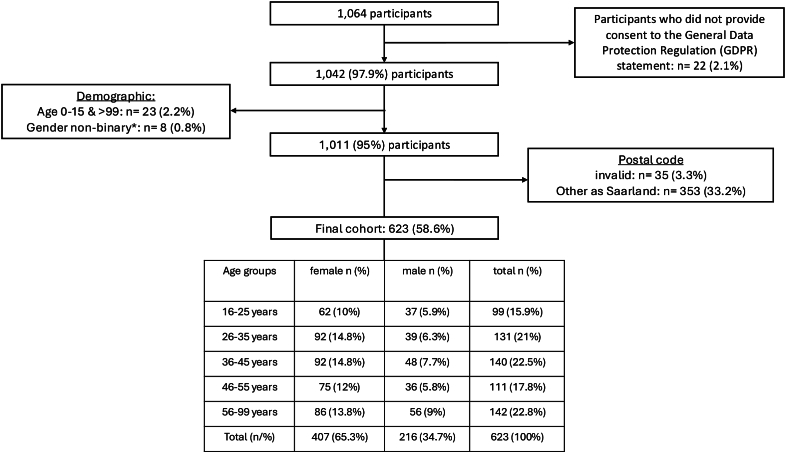
Study flowchart. The non-binary category was excluded due to an insufficient number of cases for statistical analysis The exclusion criteria comprised rejection of GDPR consent, invalid postal code, postal code from a federal state other than Saarland, and age < 16 years or > 99 years. In addition, participants who selected the non-binary gender category were excluded because the number of cases was insufficient for meaningful statistical analysis.

Inclusion criteria for the study: all participants aged between 16 and 99 with a valid postcode from the Saarland who have agreed to the data protection regulations (GDPR). Participants were categorized into five age groups: Group 1 (16–25 years), Group 2 (26–35 years), Group 3 (36–45 years), Group 4 (46–55 years), and Group 5 (56–99 years) (Fig. 1). To represent a representative cohort (95% confidence interval with a ± 5% margin of error), the data were analyzed from the Federal Statistical Office [17]. Non-binary respondents (*n* = 8) were excluded from sex-stratified analyses due to insufficient sample size. This had no impact on the overall representativeness of the dataset.

## 3 Results

### 3.1 Demographics

A total of 1058 participants completed the survey online, while an additional 6 (0.6%) participants submitted printed survey forms. The final cohort for analysis consisted of 623 (58.6%) valid and representative responses from residents of Saarland (Fig. 1).

The age distribution across the five predefined groups ranged from 15.9% to 22.8% of the total sample (Fig. 1). Within this group, the 26–35 years and 36–45 years age groups each showed the highest level of participation (*n* = 92; 14.8%). Among men, the 56–99 years age group showed the highest level of participation (*n* = 56; 9%) (Fig. 1). The group with the highest participation rate was females (*n* = 407; 65.3%).

### 3.2 Mosquito prevention: practices and perceived effectiveness

#### 3.2.1 Engagement with mosquito prevention

Regarding general mosquito prevention, 24.6% (*n* = 153) addressed the topic during a travel medicine consultation, while 50.4% (*n* = 314) engaged with it due to frequent mosquito bites. A significant sex-related difference was observed in the 26–35 age group, with men being significantly more likely than women to report no prior engagement with mosquito prevention measures despite frequent mosquito bites (*p* = 0.0015).

#### 3.2.2 Use and perceived effectiveness of protective measures

In terms of personal protective measures, wearing long clothing was the most commonly reported regular practice (36.8%, *n* = 229), followed by densely woven clothing (8.3%, *n* = 52) and permethrin-impregnated clothing (5.6%, *n* = 35). Most participants (74%, *n* = 461) agreed that wearing long clothing is effective. Around half of the participants (50.1%, *n* = 312) considered densely woven clothing to be protective. Uncertainty regarding the effectiveness of impregnated clothing was reported by 69.2% (*n* = 431) of participants (Table 1).

**Table 1:** Survey questions and gender related answers.

	n/ %		n/%		n/ %		n/%		n/ %		n/%		n/ %		n/%	
Please let us know which of the following measures you have used as a protective measure against mosquitoes?																
	Long clothing (e.g. long-legged trousers)				Impregnated clothing (e.g. with permethrin)				Particularly densely woven clothing				Removal of stagnant water accumulation in the open air/apartment			
I use it regulary	153	24.6	76	12.2	25	4	10	1.6	35	5.6	17	2.7	44	7.1	73	11.7
Never used before	32	5.1	22	3.5	**344**	**55.2**	**173**	**27.8**	**219**	**35.2**	**175**	**28.1**	**190**	**30.5**	**127**	**20.4**
Used from time to time	**222**	**35.6**	**118**	**18.9**	27	4.3	14	2.2	79	12.7	98	15.7	82	13.2	107	17.2

Please let us know which of the following measures you know of as an effective protective measure against mosquitoes?																
	Long clothing (e.g. long-legged trousers)				Impregnated clothing (e.g. with permethrin)				Particularly densely woven clothing				Removal of stagnant water accumulation in the open air/apartment			
Effective	**226**	**36.3**	**235**	**37.7**	70	11.2	80	12.8	**157**	**25.2**	**155**	**24.9**	**155**	**24.9**	**204**	**32.7**
I don't know	66	10.6	49	7.9	**230**	**36.9**	**201**	**32.3**	142	22.8	136	21.8	135	21.7	83	13.3
Not effective	30	4.8	17	2.7	22	3.5	20	3.2	18	2.9	15	2.4	32	5.1	14	2.2

Can the tiger mosquito transmit the following disease(s) when biting?																
	Malaria				Dengue				Chikungunya				Tick-borne encephalitis (TBE)			
Yes	89	14.3	76	12.2	**209**	**33.5**	**215**	**34.5**	117	18.8	130	20.9	18	2.9	17	2.7
I don't know	**146**	**23.4**	**139**	**22.3**	103	16.5	79	12.7	**180**	**28.9**	**156**	**25**	138	22.2	118	18.9
No	87	14	86	13.8	10	1.6	7	1.1	25	4	15	2.4	**166**	**26.6**	**166**	**26.6**

Table 1 presents the first part of the survey results conducted in Saarland, Germany, between February and June 2024. The symbols ♂ and ♀ denote male and female participants, respectively. Absolute values are indicated by n, and relative values are indicated by %.

The icons inserted into the table represent the participants' gender:  = female;  = male.

Participants who had attended a travel medicine consultation were significantly more likely to report regular use of long (*p* = 0.0061) and impregnated clothing (*p* < 0.0001) than those who had not.

#### 3.2.3 Knowledge and practice of source reduction

Awareness of stagnant water removal as an effective mosquito control measure was reported by 57.6% (*n* = 359) of respondents (Table 1). Despite this, the majority (50.9%, *n* = 317) had never removed standing water as a preventive measure, and only 18.8% (*n* = 117) did so regularly.

Respondents with prior training in travel medicine were significantly more likely both to recognize stagnant water removal as an effective mosquito control strategy (*p* = 0.038) and to implement this measure in practice (*p* = 0.0247).

### 3.3 Awareness of invasive mosquito species

In the section on invasive mosquito species, 64.9% (*n* = 404) of participants stated that tropical mosquito species are present in Saarland. A total of 94% (*n* = 586) had heard of *Aedes albopictus* (Table 2).

**Table 2:** Survey questions and gender related answers.

**Question**	**Yes**				**No**				**I don't know**			
	n/ %		n/%		n/ %		n/%		n/ %		n/%	
Have you already actively dealt with mosquito repellent measures before as part of a travel medical consultation?	99	15.9	54	8.7	**308**	**49.4**	**162**	**26**				
Have you already actively dealt with mosquito repellent measures? “Since I am often stung”	**224**	**36**	90	14.4	183	29.4	**126**	**20.2**				
Have you heard of the tiger mosquito?	**384**	**61.6**	**202**	**32.4**	23	3.7	14	2.2				
At what time of day do you think consistent mosquito repellent is most important in Saarland?												
• *During the day*	**188**	**30.2**	**102**	**16.4**	86	13.8	39	6.3	133	21.3	75	12
• *Time of day doesn't matter*	**166**	**26.6**	**80**	**12.8**	155	24.9	79	12.7	86	13.8	57	9.1
Do you think that the tiger mosquito poses a risk of possible transmission of pathogens to humans?	**354**	**56.8**	**168**	**27**	50	8.0	42	6.7	3	0.5	6	1

Table 2 presents the second part of the survey results conducted in Saarland, Germany, between February and June 2024. The symbols ♂ and ♀ denote male and female participants, respectively. Absolute values are indicated by n, and relative values are indicated by %.

The icons inserted into the table represent the participants' gender:  = female;  = male.

### 3.4 Knowledge of pathogen transmission

Regarding the health risks associated with *Aedes albopictus*, 83.8% (*n* = 522) of participants stated that this species could transmit medically relevant pathogens to humans. While 68.1% (*n* = 424) correctly associated the mosquito with dengue and 39.6% (*n* = 247) with chikungunya virus transmission, 26.5% (*n* = 165) incorrectly identified malaria. A share of 41.1% (*n* = 256) of the respondents claimed not to know if tick-borne encephalitis (TBE) can be transmitted by *Aedes albopictus*. (Table 1, Table 2).

When asked whether *Aedes albopictus* can transmit West Nile virus, most participants in all age groups responded with “I don't know,” totaling 366 participants (58.7%). Regarding *Borrelia* transmission, 42.9% (*n* = 267) were uncertain. For Japanese encephalitis virus, 416 participants (66.8%) indicated uncertainty. The statement “The Asian tiger mosquito cannot transmit diseases” was rejected by 80.1% (*n* = 499).

### 3.5 Perceived timing of mosquito activity

Regarding the timing of mosquito activity, 46.6% (*n* = 290) of participants identified daytime as the most critical period for active mosquito protection in Saarland (Table 2). Twilight was considered an important period by 86.7% (*n* = 540), and evening/ night by 79.8% (*n* = 497).

## 4 Discussion

This study assessed knowledge of mosquito control and invasive mosquito species in Saarland. The findings demonstrate substantial gaps in the general population's understanding of preventive measures against mosquitoes and the risks posed by invasive vectors such as *Aedes albopictus*.

The age distribution was relatively balanced across the five predefined groups, though Group 5 (56–99 years) encompasses a substantially broader age range than the remaining groups. The higher response rate among women across all age groups is consistent with previous research, which has documented a greater willingness of women to participate in surveys [18]. Especially men aged between 26 and 35 take significantly fewer mosquito prevention measures than women in the same age group, even if they are frequently bitten. It remains unclear whether this behavior is induced by the fact that they perceive mosquito bites as harmless, are subjectively bitten less often, or have a different understanding of the potential relevance [19]. This observation may also reflect previously described gender differences in health behavior, as women have been shown in other studies to place greater emphasis on preventive and healthcare-related measures [20]. From a public health perspective, this is particularly relevant, as returning travelers can serve as a source of infection even when asymptomatic due to ongoing viremia [21]. The reported prevalence of asymptomatic dengue infections varies considerably depending on factors such as study population, viral serotype, and diagnostic methods, but proportions of up to approximately 60% have been described [21].

According to the outcome of our survey, only a minority of respondents have ever attended a travel medicine consultation. Participants who discussed mosquitoes during pre-travel consultations were more likely to use long clothing and permethrin-treated garments than those who did not, indicating an association between such consultations and protective behavior. An Italian study demonstrated that individuals transfer knowledge acquired during traveling to domestic behaviors, with those who had received travel-medicine training being more likely to use insect repellent than those who had not sought pre-travel medical advice [22].

In addition, those trained in travel medicine were more likely to identify the removal of standing water as an effective mosquito control measure and implement it. Although this is not usually emphasized in pre-departure travel advice, eliminating standing water is the most effective method of preventing the establishment of invasive mosquitoes because it targets their breeding sites; repellents only offer short-term protection [23]. While over half of respondents recognized the effectiveness of removing standing water, few implemented it consistently, and nearly half never did so. This gap between knowledge and practice suggests that, although source reduction is understood conceptually, it is rarely applied, possibly due to underestimating the importance of small water accumulations or lacking awareness that this measure is particularly relevant to reduce mosquito breeding sites [24].

It became apparent that there were difficulties in distinguishing between diseases that are already present in this region, such as TBE, which is transmitted by ticks, and west nile fever, which could not be reliably identified. In addition, many respondents incorrectly associated malaria with *Aedes albopictus*, likely due to its general recognition as a well-known mosquito-borne disease. These results suggest that public awareness is influenced by media, while detailed information on transmission risks and prevention remains poorly understood.

Another notable gap concerns the timing of protective behavior. While mosquito protection is often primarily associated with evening and nighttime exposure due to the nocturnal activity of other mosquito species, such behavior is of limited effectiveness against *Aedes albopictus*, which is most active during daylight hours.

Prior exposure to vector-borne disease outbreaks is a key determinant of public knowledge and preventive behavior. Populations in regions with more frequent outbreak experience consistently demonstrate better awareness of transmission risks and protective measures [15]. Conversely, in non-endemic settings such as Saarland, misconceptions about mosquito biology and breeding habitats remain prevalent, a pattern documented across Europe and North America. In Lyon, France, 81.6% of respondents incorrectly believed that *Aedes albopictus* breeds primarily in vegetation [25], while in Germany only 11.6% could identify tree holes as larval habitats [26], and in Louisiana most participants failed to associate discarded tires with mosquito proliferation [27]. These findings highlight the need for proactive, context-specific public education that provides actionable guidance on vector control and effective protection strategies before outbreaks occur.

Limitations: This study has several limitations. There may have been selection bias, as recruitment was partly carried out via zoos and botanical gardens, which may have attracted participants with a greater interest in environmental issues. However, recruitment via public transport and television likely reduced this bias. A formal response rate could not be determined due to the distribution of the sample. Furthermore, the survey period (February–June) may have led to reduced interest in the topic, as mosquito activity increases towards summer; however, this means that the responses are more likely to reflect long-term knowledge, as intended by the study team. The questionnaire recorded behavioral patterns rather than the subjective perception of mosquito nuisance, so this must be considered when interpreting the results. Finally, the assessment of the elimination of stagnant water did not take into account differences in the participants' ability to implement this measure, although in the region studied many residents generally have access to balconies, gardens, communal green spaces or allotments that could facilitate the implementation of such measures.

The results showed that clothing that offers simultaneous protection against mosquito bites is only used to a limited extent. This highlights a substantial knowledge gap in the population regarding evidence-based protection strategies against mosquitoes. Although studies such as those by Londono-Renteria B et al. demonstrate the benefits of long-lasting permethrin-impregnated clothing [28], from a public health perspective, it is unclear which recommendations can be effectively implemented in the long term. One option would be for the public health sector to implement a set of appropriate measures as soon as the first autochthonous cases occur in this region, alongside active communication with residents.

In conclusion, the population in Saarland shows basic awareness of *Aedes albopictus* and its disease association, but important knowledge gaps remain regarding effective preventive measures and vector-borne pathogens. These findings emphasize the need for targeted public education to improve understanding of transmission risks, promote evidence-based protection strategies, and reduce the risk of mosquito establishment and outbreaks in the region.

## 5 Public health implications

The findings of this study highlight the critical importance of preparing the general population for emerging public health challenges, particularly those that are difficult to entirely prevent, such as the establishment of invasive vector species in European regions such as Saarland. As demonstrated by our results, insufficient public knowledge regarding mosquito prevention and invasive vectors can directly contribute to local outbreaks of mosquito-borne diseases in recently introduced areas, as seen in Italy, where autochthonous cases of dengue and chikungunya have been reported [4]. Addressing this issue requires a multifactorial concept [29].

From a public health perspective, it is essential to regularly reevaluate the populations knowledge of vector-borne disease prevention. Besides surveys, citizen science projects also play an important role, as they can both facilitate mosquito monitoring and enhance knowledge retention.

Germany is receptive to public health interventions, as demonstrated by a substantial increase in the prevalence of multiple healthy lifestyle behaviors between 1990 and 92 and 2008–11, indicating a positive population-level response to health promotion efforts [30]. Public education campaigns should not only communicate general preventive measures but also emphasize specific, evidence-based strategies such as the elimination of breeding sites, effective personal protection in outbreak scenarios, and the promotion of travel medicine consultations for individuals visiting endemic regions. Educational initiatives, such as illustrating the link between standing water and mosquito proliferation, should ideally begin at an early age, for instance in primary school or kindergarten, thereby fostering a lasting understanding of disease prevention within the population.

Furthermore, communication strategies should be carefully tailored to different target groups, using media channels suited to each demographic. For instance, younger people could be effectively reached through social media platforms such as TikTok or Instagram, while older audiences might be better approached through platforms like X, YouTube, or Facebook [31]. To achieve lasting behavioral change, initiatives should be repeated and strategically timed. For example, during summer months, mosquito populations typically reach their peak, coinciding with the period when most people travel abroad. This overlap may increase the likelihood of pathogen introduction, underscoring the importance of intensified awareness and preventive efforts during this time.

Another important pillar of risk minimization is the regular monitoring of mosquito populations in vulnerable areas, performed by experts. Currently, there are efforts to create risk maps that can predict where the danger might be high in the future [32]. These maps could subsequently be used to guide and prioritize mosquito surveillance activities, allowing for more targeted and efficient monitoring in high-risk areas.

While the global drivers of vector spread are not easily modifiable, targeted interventions can slow progression and create time for the development of therapeutics and vaccines [33], while ensuring populations are prepared to respond appropriately.

## Declaration of generative AI and AI-assisted technologies in the manuscript preparation process

During the preparation of this work, the authors used Perplexity AI for language refinement. All content was reviewed and approved by the authors.

## CRediT authorship contribution statement

**Kamran Dousti:** Writing – original draft, Visualization, Validation, Software, Resources, Methodology, Investigation, Formal analysis, Data curation. **Jakob Kolleck:** Resources. **Irena Harsch:** Resources. **Michael Altmoos:** Resources. **Ina Steinert:** Resources. **Mark Alexander Eichelmann:** Resources. **Phillipp Jung:** Resources. **Sören L. Becker:** Project administration, Conceptualization. **Sophie Schneitler:** Writing – review & editing, Validation, Supervision, Resources, Project administration, Methodology, Funding acquisition, Conceptualization.

## Ethics approval and consent to participate

No ethics approval was required for this study, as anonymous survey-based studies do not require ethics committee approval under German law.

## Funding statement

This project was supported through a research award granted by the “Forschungspreise der Freunde des Universitätsklinikums des Saarlandes e.V.”.

## Declaration of competing interest

The authors declare that they have no conflicts of interest.

## Data Availability

The data will be made available upon reasonable request.

## Appendix A Supplementary data

Supplementary material - Supplement 1 presents the questions of the survey conducted in Saarland, Germany, between February and June 2024.
