# Supplementary material for: Invasive mosquitoes in the non-endemic German region of Saarland: What does the public know and how does it respond?
Source: One Health. 2026 Jun 20;23:101491. doi: 10.1016/j.onehlt.2026.101491 (PMC13320451; doi:10.1016/j.onehlt.2026.101491)
Supplement: Supplementary file 1 — Supplementary material - Supplement 1 presents the questions of the survey conducted in Saarland, Germany, between February and June 2024. [file mmc1.pdf]

1. Have you already actively dealt with mosquito repellent measures before? (Yes/No)
    - Never
    - As part of a trip
    - Since I am often stung
    - As part of a travel medical consultation

2. Please let us know which of the following measures you have used as a protective measure against mosquitoes? (I use it regularly/Used from time to time/Never used before)
    - Mosquito- /fly screens
    - Eating garlic
    - Long clothing (e.g. long-legged trousers)
    - Electronic mosquito repellent (e.g. electronic vaporizers)
    - Anti-mosquito spray
    - Impregnated clothing (e.g. with permethrin)
    - Particularly densely woven clothing
    - Removal of stagnant water accumulations in the open air/apartment
    - Citronella Candles

3. Please let us know which of the following measures you know as an effective protective measure against mosquitoes? (Effective/ Not effective/ I don't know)
    - Mosquito- /fly screens
    - Eating garlic
    - Long clothing (e.g. long-legged trousers)
    - Electronic mosquito repellent (e.g. electronic vaporizers)
    - Anti-mosquito spray
    - Impregnated clothing (e.g. with permethrin)
    - Particularly densely woven clothing
    - Removal of stagnant water accumulations in the open air/apartment
    - Citronella Candles

4. What would be the impact of large-scale control of all mosquito species? (Yes/ No/ I don't know)
    - No relevant effects
    - Mosquito-borne diseases would decrease
    - The ecological balance would be disturbed

5. Is there evidence of immigrant tropical mosquitoes in Saarland? (Yes/ No/ I don't know)

6. Have you heard of the tiger mosquito? (Yes/ No)

7. Do you think that the tiger mosquito poses a risk of possible transmission of pathogens to humans? (Yes/ No/ I don't know)

8. Can the tiger mosquito transmit the following disease(s) when biting? (Yes/ No/ I don't know)
    - Malaria
    - Dengue
    - Tick-borne encephalitis (TBE)
    - Chikungunya
    - West Nile virus
    - Borrelia
    - Japanese encephalitis
    - Cannot transmit diseases

9. At what time of day do you think consistent mosquito repellent is most important in Saarland? (Yes/ No/ I don't know)
    - During the day
    - Twilight
    - Evening/Night
    - Time of day doesn't matter

I agree that this anonymous data will be scientifically evaluated. (Yes/ No)
For data protection reasons, it is not possible to subsequently assign the data you provide in the questionnaire to your personal data. The data is forwarded to us completely anonymously. The collection, storage and evaluation are carried out in accordance with the GDPR provisions. Please note that due to the anonymous data donation, it is no longer possible to view your data. We assure you that your data will be treated with absolute confidentiality and will not be made public. If, however, there is a reason to complain, you can contact the responsible data protection supervisory authority at any time: Independent Data Protection Centre Saarland – Fritz – Dobisch – Straße 12, 66111 Saarbrücken, Phone: 0681 94781-0, Fax: 0681 94781-29, Email: poststelle@datenschutz.saarland.de Participation in the survey is voluntary. Refusing to participate in this survey is possible without any disadvantages for you. The project is carried out by Saarland University, Institute of Medical Microbiology and Hygiene, Kirrberger Straße, Building 43, 66421 Homburg/Saar, phone 0 68 41 16-23900. If you have any questions about the study, please feel free to contact us at any time at tropenmedizin@uks.eu!
